# Strengthening Immunization Data: Protocol for the Evaluation of an Electronic Immunization Register

**DOI:** 10.2196/65663

**Published:** 2025-06-19

**Authors:** Meru Sheel, Cyra Patel, Gemma Saravanos, Michelle Lynch, Adeline Tinessia, Niramonh Chanlivong, Mathida Thongseng, Praveena Gunaratnam, Chansay Pathammavong, Kongxay Phounphenghack, Yongjoon Park, Sisouveth Norasingh, Dheeraj Bhatt, Nyambat Batmunkh, Marcela Contreras, M. Carolina Danovaro-Holliday

**Affiliations:** 1 Sydney Infectious Disease Institute Faculty of Medicine and Health University of Sydney Sydney Australia; 2 School of Public Health Faculty of Medicine and Health University of Sydney Sydney Australia; 3 National Centre for Epidemiology and Population Health Australian National University Canberra Australia; 4 Susan Wakil School of Nursing and Midwifery Faculty of Medicine and Health University of Sydney Sydney Australia; 5 Clinton Health Access Initiative Vientiane Lao People's Democratic Republic; 6 Ministry of Health Vientiane Lao People's Democratic Republic; 7 Vaccine-Preventable Disease and Immunization Unit World Health Organization Vientiane Lao People's Democratic Republic; 8 UNICEF Vientiane Lao People's Democratic Republic; 9 Special Program Comprehensive Immunization Pan American Health Organization Washington, DC United States; 10 Department of Immunizations, Vaccines and Biologicals World Health Organization Geneva Switzerland

**Keywords:** electronic immunization register, evaluation, process evaluation, digital technology, vaccination coverage, health system strengthening, mixed methods, data quality

## Abstract

**Background:**

Electronic immunization registers (EIRs) can strengthen immunization systems and, if used effectively, can lead to greater efficiency and improvements in vaccination coverage. Several low- and middle-income countries introduced EIRs for COVID-19 vaccination and are now integrating them for routine immunization.

**Objective:**

This study aims to describe the protocol used to evaluate the implementation of an EIR in the Lao People’s Democratic Republic in 2022. In addition, it seeks to identify opportunities to improve implementation, scale-up, and sustainability in the country.

**Methods:**

To evaluate the implementation of the EIR in the Lao People’s Democratic Republic, we will (1) map the EIR workflow process, (2) examine EIR user and stakeholder perspectives, and (3) assess the EIR data quality. Data will be collected and analyzed through a mixed methods approach. This evaluation will involve a document review, observation of workflows in health facilities, health facility user surveys, key informant interviews with decision makers, and an assessment of immunization data quality. This protocol details the methods for each of these components. The evidence generated will be triangulated to identify the strengths and weaknesses of the early implementation phase of the EIR, facilitators of and barriers to the implementation, and whether the introduction of the EIR has improved immunization data processes and quality compared with paper-based processes.

**Results:**

Data collection took place between April 2024 and August 2024. Data analysis is currently ongoing, with results expected to be shared with study stakeholders in October 2024.

**Conclusions:**

This early evaluation will contribute to developing a road map for the nationwide strengthening and sustainability of the EIR based on challenges and lessons learned, potentially streamlining further implementation efforts and enabling more effective use of the EIR. This paper presents a methodology for evaluating EIR implementation that can be replicated in other low- and middle-income countries implementing EIRs.

**International Registered Report Identifier (IRRID):**

DERR1-10.2196/65663

## Introduction

### Background

Vaccines are a highly effective public health intervention, with childhood vaccination programs estimated to have prevented as many as 154 million deaths over the past 50 years [1]. Despite significant improvements in coverage of routine immunization over the past 2 decades, there have been challenges in recent years, as evident from the coverage of the third dose of diphtheria-tetanus-pertussis (DTP) vaccine plateauing at approximately 86% [2]. Coverage of other childhood vaccines has increased over time, but many vaccines remain underused [3]. Widespread disruptions to routine immunization programs during the COVID-19 pandemic and consequent declines in coverage [4,5] meant an additional 5 million children missed receiving a single dose of DTP vaccine in 2021 [4]. Urgent recovery efforts are needed to prevent an estimated 49,000 excess deaths by 2030 [6,7].

Global immunization actors such as the World Health Organization (WHO), United Nations Children’s Fund (UNICEF), and the World Bank have promoted the implementation of digital tools for health, which include electronic immunization registers (EIRs), to improve vaccine uptake and management of immunization programs and to strengthen health systems [8,9]. An EIR is a confidential, computerized, population-based routine system used to capture, store, access, and share individual-level data on vaccine doses administered (Multimedia Appendix 1) [10]. An EIR is a component of a comprehensive health information system that consolidates individualized immunization information for various purposes, such as vaccine coverage monitoring, safety monitoring, and supply management [11] (Table 1). EIRs have the potential to improve vaccination coverage by enhancing the monitoring of individuals’ immunization status, automating the tracking and recalling of defaulters, identifying health care providers with lower performance, and providing decision support for immunization [12-14]. In addition, EIRs can reduce costs to the health system [15,16]. From a policy and governance perspective, EIRs allow countries to identify coverage gaps and inequities at the national, subnational, and local levels, facilitating evidence-based decision-making and developing targeted initiatives to address gaps [17]. The use of EIRs aligns with a key priority of the Immunization Agenda 2030 (IA2030), the strategy guiding improvements in immunization programs globally, to use high-quality data to track progress and inform decision-making at all levels of the health system [18]. Furthermore, when EIRs are linked with other data as part of the broader immunization information system, they can strengthen safety monitoring and be used to conduct vaccine effectiveness studies in almost real time [19-26].

**Table 1:** Definitions of terms.

Term	Definitions	Characteristics
EIR^a^	It is a confidential, computerized, population-based routine system to capture, store, access, share, and consolidate individual-level data on each vaccination. Immunizations are recorded directly into an electronic system. Immunization actors at other levels of the health system (ie, district, province, and national) can directly access the data.	At a minimum, a register typically includes an individual identification number or code, names, date of birth or age, sex or gender and information on the vaccine administered (vaccine type and brand and date of administration) and the vaccine administration itself (date and facility or health care provider). Data are recorded and stored electronically (online or offline) rather than on paper^b^. Ideally, registration occurs as close as possible, in time and place, to the vaccination act itself. Individuals may be identified using a unique identifier (eg, a national identification number, biometrics, or a combination of personal information). Data for each individual (improving population denominator data quality). It is accessible at all levels of the health system^b^. Data aggregation and visualization are automated, with aggregation by various factors (eg, age, sex, geographical region, and other factors for which data are captured) possible with relative ease^b^. Facilities can conduct timely individualized follow-up for vaccination^b^. It includes processes for data security and patient confidentiality^b^. Linkage, interoperability, and integration with other immunization, health, and civil registry information systems are possible^b^. It can allow individuals to access their vaccination history and generate a record of their vaccinations.
Paper-based immunization systems	These are paper-based individual-level records of vaccinations administered to a defined target population. Immunizations are typically recorded in a paper-based register held by the health facility where the vaccine was administered. A vaccination card or other home-based record or tickler files (a systematic way of organizing vaccination cards to enable follow-up of children due for vaccination) may also be used to record the immunization. Health workers use tally sheets to count and consolidate the number of vaccines administered over a given time (usually a month) and then report to the district, provincial, or other subnational level office. Subnational offices compile multiple reports and report up to the health system, until they reach the national office.	Data are recorded manually, that is, typically with pen and paper. Typically, individual-level data are accessible only by health facilities. These systems might rely on updated information or data from child health records. Data aggregation is manually completed by health workers recording the number of vaccines administered on a tally sheet, often for reporting purposes. Only limited information is captured, typically the number of specific types of vaccines administered by age group and gender. Data visualization is limited; aggregated data are typically recorded in a monthly summary form after being counted manually from tally sheets. Individualized follow-up (recall) may be challenging, and it requires staff to manually review handwritten registers, as the data are not easy to retrieve. Data may be recorded in multiple registers over the years, and it is a manual, labor-intensive process that is prone to errors. There is also a risk of loss of data from being physically displaced or fading ink. Linkage or interoperability with other data systems is not possible.
Immunization registry system	It encompasses the entirety of the ecosystem of processes, resources, regulations, technology, and infrastructure needed to support the use of an EIR to enable improved efficiency and improved vaccination coverage.	It includes all health system structures necessary for an effective EIR, such as human resource capacity, IT and digital infrastructure, financial resources, governance and policy framework, and mechanisms for effective data use and evidence-based decision-making.
Immunization information system	It is a comprehensive health information system (encompassing multiple information sources) that consolidates individualized immunization information for various purposes, including, but not limited to, vaccine coverage monitoring, safety monitoring, supply management, and disease surveillance.	Individual-level information on vaccination and vaccination events is available. It encompasses all systems involved in capturing vaccine uptake, calculating vaccination coverage, monitoring adverse events, conducting public health surveillance of vaccine-preventable diseases, managing vaccine and related commodity supply and stock, and potentially tracking the behavioral and social drivers of vaccination. Individual systems may be interoperable or linked to support functions, such as safety monitoring, stock management, disease surveillance, or outbreak response, and with civil registry systems, other health information systems, and other population-based data systems to enable detailed vaccine effectiveness and safety analyses in specific populations. It may include a communication platform that allows for targeted communication with health care professionals and the public. It links to other electronic technologies, such as decision support systems for health care providers (eg, automated alerts to physicians for vaccination catch-up).

^a^EIR: electronic immunization register.

^b^These are essential features of EIRs based on information extracted from multiple sources [11,27-32].

There is a growing body of evidence that EIRs can strengthen immunization programs in low- and middle-income countries (LMICs) by enabling greater data use [33,34]. A 2015 EIR evaluation in a province of Vietnam found that full vaccination coverage of children aged <1 year improved from 75.4% to 81.7% following the province-wide introduction of the EIR along with an SMS text message reminder system and to 99.2% one year after the intervention [35]. In Pakistan, an EIR with decision support and SMS text message reminders, supplemented by health worker attendance logging and a web-based dashboard, increased polio vaccination coverage from 12% in May 2019 to 97% in December 2019 [36]. However, the attribution of improvements in coverage directly to EIRs is problematic, and several studies have failed to detect an effect or an impact [37-39]. Moreover, little is known about the level of investment needed for the establishment and ongoing maintenance of EIRs in relation to the benefits achieved.

The unprecedented scale and speed at which COVID-19 vaccination programs were rolled out prompted several LMICs to implement EIRs for the first time. While many of these systems were designed for COVID-19 vaccination and were frequently motivated by the need for vaccine certificates and monitoring coverage, the positive experiences have encouraged countries to invest in efforts to integrate and scale up EIRs into routine immunization programs [7,40]. Well-designed evaluations at critical time points are necessary to ensure robust implementation, assess the usefulness of the system in guiding policy and practice, monitor coverage, and help prioritize allocation of resources. Few EIR evaluations have been conducted to date [41], and there are limited assessment protocols that are context specific and co-designed with key stakeholders and technical experts. Moreover, while there are standard methodologies to evaluate public health surveillance systems [42], systematic frameworks or approaches to evaluate EIRs, particularly in LMICs, are lacking [43].

### Immunization in the Lao People’s Democratic Republic and Implementation of an EIR

The national immunization program of the Lao People’s Democratic Republic (Lao PDR) provides funded vaccination for 12 vaccine-preventable diseases. The vaccines are as follows: Bacillus Calmette–Guérin vaccine; hepatitis B vaccine; oral poliovirus vaccine; inactivated poliovirus vaccine; the pentavalent vaccine containing diphtheria, pertussis, tetanus, hepatitis B, and *Haemophilus influenzae type B*; pneumococcal conjugate vaccine; measles-rubella vaccine; Japanese encephalitis vaccine; tetanus-diphtheria vaccine; seasonal influenza vaccine; and human papillomavirus vaccine (Multimedia Appendix 2).

Despite significant investment into immunization programs in the Lao PDR, the COVID-19 pandemic impacted routine vaccination coverage with significant declines in 2020 and 2021. The estimated coverage of DTP3 and the first dose of measles-containing vaccine in 2022 was 92% and 82%, respectively [44]. Challenges to the immunization program include identification of unvaccinated (zero-dose children) and partially vaccinated children, the need to reduce dropout rates between doses, improving vaccination timeliness, sustainable delivery of immunization services in hard-to-reach communities, timely recording and reporting of immunization data, challenges in attaining appropriate denominators, and the accuracy of coverage estimates [45].

Currently, the Lao PDR immunization information system uses a combination of paper-based and digital systems. Since 2015, the Lao PDR Ministry of Health (MoH) has used the District Health Information Software 2 (DHIS2), an open-source digital health information management system, to manage information on malaria, tuberculosis, HIV, and the immunization program. Vaccination coverage statistics were generated from this system using aggregated data collected from health facilities, limiting the ability to appropriately evaluate the immunization program’s performance and identify where vaccination coverage is low. This limitation also affects the measurement of key indicators, such as dropout rates, which require individual-level data for accurate estimation.

During the COVID-19 pandemic, the Lao PDR the Maternal Child Health Centre (MCHC) of the MoH established a custom EIR comprising of an individualized immunization system using the DHIS2 Tracker software to capture individual COVID-19 vaccination data nationally. This system was designed to be used in tandem with DHIS2 as a health information management system [46]. On the basis of the lessons and experiences learnt from the development and implementation of the COVID-19 Vaccination Registry, a separate EIR for childhood immunization targeting children aged <5 years was developed. This EIR was piloted in November 2022 in a district hospital in the capital city and subsequently rolled out across 2 provinces between May 2023 and August 2023. As of January 2024, health workers in all 18 provinces had received training on the use of the EIR. Currently, the system only captures vaccination data for children aged ≤5 years. Through this transition phase, health facilities are required to use both the EIR and the traditional paper-based form for recording and reporting purposes at fixed and outreach immunization clinics.

### Rationale and Aims

Early and ongoing evaluations provide valuable insights into the current strengths and challenges of the system, its implementation, and barriers to and facilitators of the successful transition from an aggregated paper-based system to a digital system. Information arising from evaluation can be used by decision makers to address challenges during the early stages of implementation of the EIR. The data can inform system-related updates and address human resource needs and requirements to make the system sustainable.

This study aimed to conduct an evaluation of the Lao PDR EIR for childhood immunization to identify opportunities for improving and strengthening its implementation, scale-up, and sustainability. The specific objectives were as follows:

1. To describe and map the components and workflow processes of the immunization registry system
2. To examine the perspectives of those involved in implementing and using the system, namely health system decision makers and health workers at health facilities, to determine the extent to which the system is fit for purpose
3. To assess the quality of the data captured in the EIR
4. To examine the barriers to and enablers of implementation and long-term use of the system.

This pilot study was designed to provide recommendations to the Lao PDR MoH and inform EIR implementation and scale-up.

## Methods

### Conceptual Framework

This evaluation is underpinned by the theory of change approach, which theorizes that the implementation and use of an EIR can lead to improved immunization outcomes. A theory of change for supporting data-informed decision-making for immunization programs, arising from a realist review of interventions aimed at improving the use of immunization data, has been adopted for this evaluation [33]. This theory of change hypothesizes that effective implementation of an EIR will translate into improved data use, which, in turn, will drive demand for data quality and access, building a culture of data use to drive decision-making at all levels of the system. In the longer term, increased data use is expected to lead to improvements in vaccination coverage and reductions in the burden of vaccine-preventable disease.

However, this theory of change may be erroneously interpreted as a linear, organic process following the introduction of an EIR, while, in reality, the environment within which the EIR is implemented affects its success or failure. The IA2030 Data Action Theory of Change Framework partly reconciles this and identifies digital tools as 1 of the 5 building blocks needed to improve the availability and fit-for-purpose data, increasing data use in decision-making and resulting in improved program outcomes [47]. The other four blocks include (1) establishing a continuous cycle of assessment and quality improvement, (2) having clearly defined governance processes and policies for data collection and use, (3) empowering health workers to use data for decision-making, and (4) documenting ways to improve data and its use [47]. While more holistic, the IA2030 Data Action Theory of Change Framework depicts the EIR as one of several inputs rather than as a health system-strengthening intervention that is implemented within an evolving environment. The IA2030 Data Action Theory of Change Framework and the theory of change framework developed by Osterman et al [33] have both been adopted as underpinning conceptual frameworks in this evaluation.

In this study, we will examine the “immunization registry system,” which incorporates the EIR and the ecosystem surrounding it. The term “EIR” refers to the computer-based tool used to capture, store, access, and share individual-level data on vaccine doses administered [27]. The immunization registry system encompasses the entirety of the processes, resources, regulations, technology, and infrastructure needed to support the use of an EIR, enabling improved efficiency and improved vaccination coverage (Table 1). Mapping of the Lao PDR immunization registry system was first performed through a document review using the Pan American Health Organization criteria for EIR evaluation [43].

**Figure 1:**
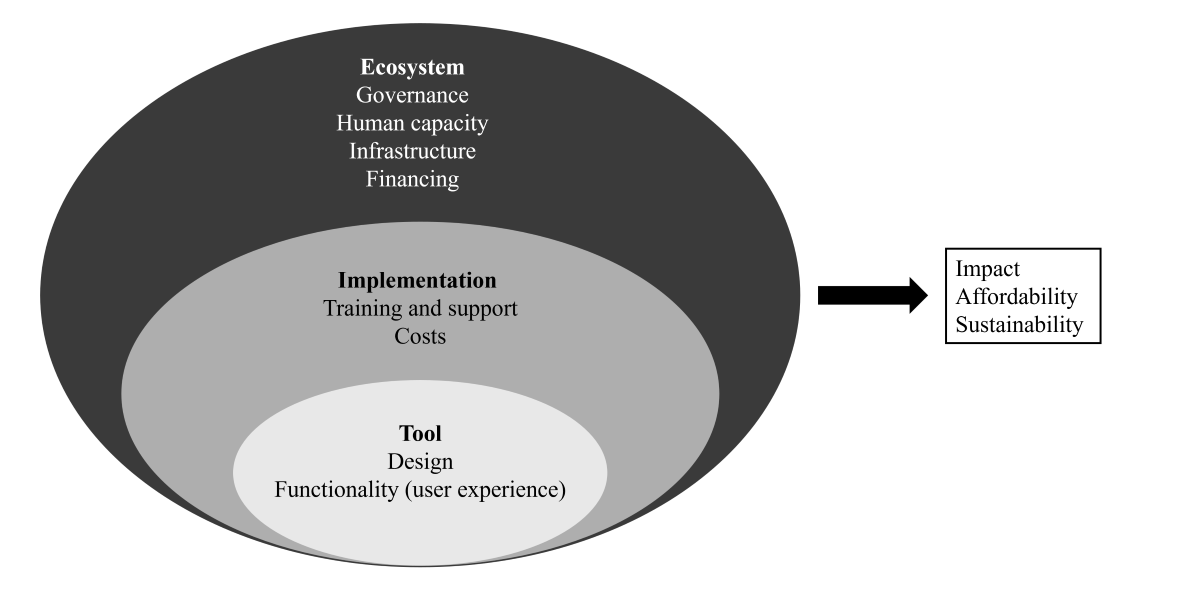
Framework for the evaluation of the Lao People’s Democratic Republic electronic immunization register (adapted from report published by the Bocconi School of Management [28]).

For primary data collection, we adapted a recently published framework for the evaluation of EIRs and electronic logistics management systems (Figure 1) [28]. This approach focuses on the design and function of the tool, its implementation, the contextual factors affecting implementation (ie, the ecosystem), and the impact and sustainability of the tool. The framework incorporates the EIR’s impact on immunization service delivery and its financial sustainability and viability as outcomes. However, it is important to note that achieving these outcomes may take many years, and while an EIR is a critical tool, much like a “vector,” implementation of an EIR is unlikely to realize these benefits without addressing other reasons for poor uptake of vaccination. As the Lao PDR EIR has only recently been implemented, our evaluation focuses on the uptake and use of the EIR, particularly its acceptance and use by decision makers and health workers.

### Study Design and Data Collection

#### Overview

**Figure 2:**
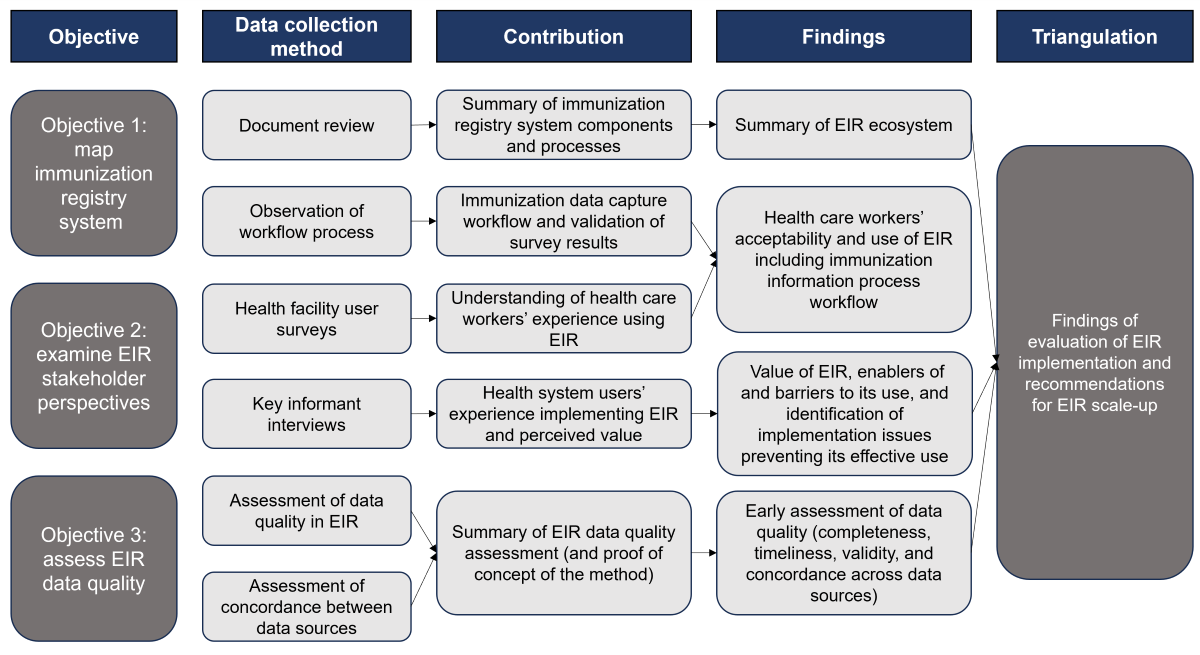
Outputs from each study component and contribution to the summary of findings from this process evaluation of the Lao People’s Democratic Republic electronic immunization register (EIR).

We will use quantitative and qualitative research methods to (1) map the Lao PDR immunization registry system components and workflows, (2) examine the EIR stakeholder perspectives, and (3) assess EIR data quality. The evaluation methods and data collection tools were informed by a review of the key literature on the use and evaluation of EIRs, in addition to evaluation protocols shared by experts working in the field. This mixed methods approach will be sequential and iterative, whereby the outcomes of one component will inform subsequent elements of the study (Figure 2).

Data collection methods will involve (1) a document review, (2) observation of workflows in health facilities, (3) health facility user surveys, (4) key informant interviews with decision makers, and (5) an assessment of immunization data quality (Table 2).

**Table 2:** Summary of data collection methods, target population, and analysis methods for this evaluation.

Activity	Target population	Sample strategy and size	Analysis
Document review	Documents pertaining to the performance and operation of the Lao PDR^a^ EIR^b^	N/A^c^	Narrative synthesis based on the criteria by PAHO^d^ for EIR evaluation [44]
Observation of the workflow process	Health facilities providing immunization services	4 purposively selected health facilities	Narrative summary and validation of health facility surveys
Health facility surveys	Health facility immunization providers	24 randomly selected community health facilities and 2 purposively selected large hospitals (26 facilities in total)	Descriptive statistics summarizing quantitative and qualitative data
Key informant interviews	Decision makers and implementing partners at the Lao PDR Ministry of Health and other partner agencies working in immunization and digital system teams at the national and subnational levels	12 to 14 purposively selected participants	Thematic analysis
Data quality assessment	All health facilities using the EIR during the agreed period of analysis	4 purposively selected health facilities (concordance) Cohort of children who were born in 2023 and have their records available in the EIR (selected data and immunization quality indicators)	Metrics to quantify the concordance of data in the EIR compared to existing methods of data capture Metrics to quantify the completeness, timeliness, and validity of the data in the EIR

^a^Lao PDR: Lao People’s Democratic Republic.

^b^EIR: electronic immunization register.

^c^N/A: not applicable.

^d^PAHO: Pan American Health Organization.

#### Method 1: Document Review

We will search for peer-reviewed publications, gray literature, and internal government documents that pertain to the performance and operation of the Lao PDR EIR, the national immunization program, the health system, or the governance of health and immunization systems. Documents detailing policies and procedures (eg, organizational charts, immunization agenda goals or strategies, recent evaluations of immunization system, regulatory frameworks, and standard operating procedures), regulations, national immunization technical advisory group’s recommendations, immunization schedules and indicators, technical reports and guidance documents, digital and paper-based tools, and educational resources will be considered for inclusion. We will extract data based on the criteria for EIR system evaluation developed by the Pan American Health Organization [30], with domains, including system scope, normative and legal context, system architecture, maintainability and sustainability, human resources, modules included in the system, and functionalities.

#### Method 2: Observation of the Vaccination Workflow

We will undertake health facility visits in 4 purposively selected sites to observe the vaccination process and how these intersect with data capture, entry, and reporting. This activity will be a proof of concept, conducted in a high-performing and a low-performing district based on their vaccination coverage and as advised by local in-country stakeholders. This activity is designed to support an accurate understanding of data capture and process flow, provide further context to variability between health facilities and service types, and provide validation of other data collected. A data enumerator with experience in clinical practice, including immunization, will observe how data are recorded by health facility workers during 3 vaccination encounters at each facility. Observations will be recorded using a site observation tool (Multimedia Appendix 3) and will capture a mix of quantitative and qualitative (free text) items.

#### Method 3: Health Facility User Surveys

We will examine the perspectives of EIR users at health facilities using a cross-sectional survey adapted from existing tools (Multimedia Appendix 4; Pan American Health Organization, unpublished report, May 2025). Survey questions are structured into six key domains: (1) process, (2) infrastructure, (3) data management and use, (4) workforce, (5) user satisfaction with the EIR, and (6) user perception of EIR data quality. The user satisfaction tool covers the user’s perceptions of the ease of using the EIR, its efficiency compared with paper-based methods, data security, impact on immunization service delivery, and overall satisfaction with the EIR. The tool measuring users’ perception of information quality covers the user’s satisfaction with the accuracy, completeness, timeliness, ease of access, and usefulness of data in the EIR. We iteratively tested and revised the survey instrument based on feedback, then translated it into Lao, where it was tested by technical expert native speakers. We will conduct a pilot of the survey at 2 to 3 health facilities to test the tools and make final adjustments to the data collection tools. Data from the pilot study will be included in the final dataset. Trained data enumerators with experience in immunization and surveys will administer the survey using the REDCap (Research Electronic Data Capture; Vanderbilt University) system. The REDCap mobile app was selected for this study as it can support secure offline data capture on mobile devices, critical for data collection in remote areas with poor internet connectivity [48,49].

#### Method 4: Key Informant Interviews

There is a lack of consensus on how to quantitatively measure data use for immunization, with indicators generally being poorly defined, lacking specificity, and failing to capture the complexity involved in assessing the extent to which data are used. At present, a qualitative approach is more appropriate to understand facilitators of and barriers to the implementation, how data are used, and how the ecosystem and digital tool impact data use. We will use a semistructured interview guide informed by previously published literature and protocols provided by content experts (Pan American Health Organization, unpublished report, May 2025) [50]. The interview guide will cover seven domains: (1) overall perceived net benefits of the EIR, (2) implementation process of the EIR, (3) impact of the EIR on the national immunization program, (4) impact of the EIR on decision-making, (5) the cost of implementing the EIR, (6) linkage and interoperability with other health data systems to support program evaluation, and (7) investment in the EIR. This will evolve based on findings from user surveys. In total, 2 to 3 members of the study’s advisory group from the Lao PDR will translate and pilot the tool in Lao. Interviews will be reviewed by the study team in real time for quality assurance and to ensure that interviews continue until saturation is reached. Thematic analysis will be used to interpret and report patterns within the data.

#### Method 5: Assessment of Immunization Data Quality

A comprehensive assessment of immunization data quality typically involves an audit of paper and electronic records. This activity can be resource intensive and is challenging when records are missing or difficult to retrieve, such as paper-based records and child home-based documentation. Nonetheless, a data quality assessment offers a powerful way to quantify the impact of introducing digital tools and demonstrate improvement in the short term. A comprehensive audit of immunization records across all facilities included in this study is not feasible; however, we will conduct a targeted audit to test the proof of concept, drawing on several established audit methods.

First, we will assess data concordance, that is, agreement of different sources of immunization data (refer to the data concordance tool in Multimedia Appendix 5). This will be undertaken in 4 purposively selected health facilities, with 1 site including the district-level hospital. Data enumerators will collect aggregate data on selected indicators (hepatitis B birth dose, DTP-containing vaccine [pentavalent vaccine] dose 1, and measles-containing vaccine dose 1) over a 3-month period. Counts will be compared across paper-based health facility registers, monthly reports, and EIR records.

In addition, we will obtain an EIR dataset and assess the data quality of the records of the cohort of children born in 2023. We will use indicators from existing frameworks (Pan American Health Organization, unpublished report, May 2025) [51] to assess the data quality dimensions of completeness (absence of missing data), timeliness (recorded within a specific time frame), and validity (absence of errors).

### Study Location, Sampling, and Study Participants

This study will be conducted in Vientiane capital and Vientiane province, where the EIR was first implemented in 2022. We purposively selected 1 district hospital in Vientiane capital and the provincial hospital in Vientiane province. In addition, a target of 50% of all health facilities will be selected while still capturing a sufficiently large number that would provide critical data. We will randomly select 24 (39%) of the 61 health facilities in Vientiane province, including both health centers and district hospitals. To do so, we will apply stratified random sampling proportionate to the number of health facilities in each district to identify a sample of health facilities to survey. To reduce selection bias and ensure a geographically representative sample, we will use random sampling proportional to population size, ensuring that health facilities from all districts are included. At each health facility, any health worker who vaccinates or uses the EIR to collect or review immunization data will be targeted for recruitment.

We will select 4 (15%) of the 26 sites for the site observation and data quality assessment components of this study. We will purposively select these sites to capture diverse data from different health facilities. We will use coverage estimates from 2023, using data from the aggregated DHIS2 database, to identify districts with high and low vaccination coverage, selecting 2 facilities each from a high- and low-performing district. We will only select sites that provide outreach services to allow us to assess whether data quality differs by fixed or outreach services. Local study investigators and technical advisors will provide advice and guidance on the selection of facilities.

For face-to-face key informant interviews, we will aim to recruit 12 to 14 stakeholders. Stakeholders will be purposively selected by the study team and the technical advisory group for inclusion in the study. Stakeholders will represent different functions and roles in the health system, with knowledge of the roles in immunization program planning and decision-making, including members of the country’s National Immunization Technical Advisory Group [52]. Stakeholders will also include those involved in implementing the EIR, such as individuals at the subnational level. In case of unavailability, stakeholders will be asked if there are others who may be able to contribute to the study (ie, snowballing). We will also interview stakeholders from key partner organizations, such as the WHO; UNICEF; and Gavi, the Vaccine Alliance, who are involved in using immunization data for programmatic and monitoring purposes.

An in-country coordinator was recruited to the study team to better facilitate recruitment and participation. The in-country coordinator will seek permission from each of the relevant provincial and district health center staff members from the preselected health centers and key informants before commencing study activities. Prior permission to attend the health facility and collect data from the site will be obtained from the immunization lead at the MCHC at the MoH. Consent will be sought from both the health worker being surveyed and the person in charge of supervising routine vaccine activities or the person in charge of immunization data management. All individual participants will be provided with a participant information sheet in English or Lao before the survey or interview, with written consent collected at the time of the survey.

### Study Management and Conduct

#### Governance and Study Monitoring

A technical advisory group comprising experts from the MCHC, the department of finance and planning of the MoH, the WHO Country Office, UNICEF, and the University of Health Sciences of the Lao PDR was invited to provide advice and guidance on the design of the evaluation. The advisory group includes in-country representatives who are involved in the implementation, training, monitoring, and maintenance of the EIR. All study materials will be reviewed by the advisory group to ensure they are context appropriate.

#### Data Management

Health facility surveys, on-site workflow observations, and key informant interviews will be conducted in Lao by local research team members who have knowledge of the national health and immunization system of Lao PDR. All data enumerators will receive training before data collection. Training sessions will be developed and delivered by study coordinators with expertise in quantitative and qualitative research methods. Training topics will include public health research methods, research ethics, interview techniques, data collection and management, and troubleshooting of data collection tools.

Survey data will be captured digitally in the REDCap mobile app. Interviews will be digitally recorded and then transcribed by native language speakers. Workflow observation and immunization data from records held at health facilities will be captured on paper and later transcribed into a secure digital format. All records will be held securely by the local study team for at least 5 years.

#### Data Quality

All data collection tools will be pilot-tested before the start of fieldwork. During fieldwork, survey and observation data will be monitored daily for gaps, inconsistencies, and clarity of free-text responses. This real-time feedback between data enumerators and field coordinators will support timely modification of the data collection tools, troubleshooting, correction and clarification of data, and additional training of data enumerators as required.

All data obtained from the EIR, including both individual and aggregated data on vaccination coverage, will be deidentified before acquisition. While some analysis may be completed at the health-facility level, caution will be taken to ensure that reporting of facility-level results does not adversely impact the facilities or any individual’s reputation or standing with local organizations in the Lao PDR. Therefore, results by individual facility may not be published; however, summary findings on factors associated with improved data quality or workflows will be reported.

### Ethical Considerations

The ethical conduct of this study was reviewed and approved by the Lao PDR MoH Institutional Review Board (National Ethics Committee for Health Research; submission ID 2023.71) and the University of Sydney Human Research Committee (protocol 2024/038). Protocol amendments will be reviewed by both ethics committees. Participants will be provided with a participant information sheet and asked to give written informed consent, with the option of opting out at any time. Data will be anonymized and deidentified. No compensation will be provided for participants.

### Outcomes and Analysis

Findings from study components will be separately analyzed and then triangulated to develop a summary of the immunization registry system with a focus on EIR operations and integration with other key systems.

The document review will provide contextual background for analyzing the study findings, including information about process workflows and the health and digital environment in which the EIR is being implemented. Both the health facility user surveys and data quality assessment will be quantitatively analyzed, with results reported as descriptive statistics. For the user experience and perspectives from the survey, we will report on current practices with regard to recording immunization data, enablers of and barriers to using the EIR, and user perceptions of and satisfaction with the EIR. Survey results will be reported as counts and proportions with 95% CIs for each survey item (eg, the number and proportion of respondents who were very satisfied, satisfied, unsatisfied, and very unsatisfied with the completeness of EIR data). Findings from the site observations will be narratively summarized and used to verify findings from the survey and document review.

Key informant interviews will be transcribed into Lao and translated into English for thematic analysis, with themes identified inductively. Themes will be discussed with the research team and modified until an agreement is reached.

For the data quality assessment, we will assess data for the following:

- Completeness of EIR records, that is, absence of missing records or data elements (eg, proportion of records in the EIR with missing date of birth) and completeness of the EIR (eg, proportion of births registered in the EIR)
- Timeliness, that is, data are recorded within predefined time frames (eg, proportion of vaccination events recorded within 3 days of vaccination as per standard operating procedures)
- Validity, that is, data are accurate and do not contain errors (eg, the proportion of records with the date of vaccination before the date of birth)
- Concordance, that is, agreement of different sources of immunization (eg, the number of hepatitis B birth dose vaccinations recorded in the health facility report, district level report, and EIR for a given month).

Subgroup analysis will include sex, location, and service type (particularly fixed vs outreach services). Summary statistics from the data quality assessment will be reported as counts or proportions.

We will triangulate the results across the 5 study components using the framework derived inductively based on themes from the key informant interviews. Key findings and their interpretation from each study component will be mapped and tabulated by theme and then compared across each theme to identify convergent and divergent findings. Through this process, we will identify the strengths and weaknesses of EIR implementation; the facilitators of and barriers to implementation; and whether the introduction of the EIR has improved immunization data flow, processes, and quality compared with paper-based processes. This will then be used to codevelop recommendations, in collaboration with the Lao PDR MoH, on how the nationwide scale-up of the EIR can be implemented more effectively.

## Results

### Overview

Participant recruitment for this study began in April 2024 and concluded in July 2024. We surveyed workers at 26 health facilities and interviewed 18 key informants. We collected data on administered vaccinations from 4 sites for our assessment of data concordance concurrently during the health facility surveys and subsequently obtained data from the EIR for the 2023 birth cohort in August 2024. Data analysis is currently ongoing.

### Dissemination

Results will be shared with key stakeholders, including the Lao PDR MoH and study funders, before public dissemination. Study findings will be presented and collaboratively reviewed with the advisory group and key stakeholders from the Lao PDR during a result dissemination meeting in October 2024. The meeting will be used to review and draft recommendations for improving the nationwide enhancement of the EIR. After finalizing the results, we will summarize our findings in a formal report, brief the MoH, and prepare the manuscript for submissions to peer-reviewed publications. Results will be shared with the key stakeholders, including all study participants and external organizations.

## Discussion

### Anticipated Findings

The shift from manual methods of data collection and reporting to electronic methods offers opportunities to improve the quality, availability, and use of immunization data, leading to greater accountability and ownership of data [33,53-55]. The use of the health management information system DHIS2 in Nigeria allowed users at different levels of the health system to track progress toward goals in real time, understand areas of poor immunization performance, and provide appropriate support to improve performance [55]. Cost savings, driven largely by reductions in the amount of time spent by health workers on immunization reporting due to the use of an EIR, have been reported in Tanzania and Zambia. Additional savings were attributed to improved vaccine management (eg, fewer trips for vaccine resupply) and reduced printing costs [16]. Health workers in facilities in Tanzania reported saving >70 hours each year that they could allocate to patient care [56]. Increased data visibility led to improved practices for ensuring data quality, resulting in fewer errors and greater data completeness [56].

While there are clear benefits of implementing EIRs, the shift from paper-based systems to digitized immunization information systems poses new challenges surrounding implementation, uptake, and sustainability [16,57]. In the Lao PDR, the switch to digitized systems, such as DHIS2, has been underway for the last decade and is still ongoing [58]. EIR implementation is resource intensive, and poor implementation can result in a waste of resources. For example, the proliferation of noninteroperable platforms used to collect and manage data in West Africa during the 2014 to 2016 Ebola virus crisis resulted in siloed and duplicated information systems that were detached from national systems [59]. Limited workforce capacity to manage and use the data and technical constraints, such as unreliable electricity, battery life, and network coverage, led to several tools being abandoned after the acute crisis [59]. This led to a waste of time and financial resources as well as missed opportunities for strengthening the health system. Similar challenges were identified in the implementation of an EIR in Tanzania, where workforce capacity constraints on both time and technical competencies; the use of other electronic systems in parallel with the EIR; and infrastructural challenges, such as inadequate computing equipment, internet, and electricity outages, hindered the implementation and use of the EIR [60]. At present, the Lao EIR is not used for automated reminders for caregivers, but this is an area to be explored in the future.

Similar to other health system strengthening initiatives, the impacts of new interventions (technological or other) are often realized several years after their implementation and are difficult to attribute to the technology, given the other system factors that also affect the outcome of improved vaccination coverage. Furthermore, there is a false perception that the implementation of a digital innovation alone is sufficient to bring about improvements in vaccination coverage, but this is not the case. EIRs can only be an effective health system strengthening tool if the information system works synchronously, with an appropriate enabling health digital environment including infrastructure and regulatory framework; ongoing resourcing including human, financial, and technological investments; strong leadership to advocate for their implementation, procedures, and use; and ongoing monitoring and evaluation [61]. Achieving EIR scale and sustainability requires time, investment, and adaptations based on the local context [10]. Often, there can be challenges with user experience resulting in compromised data quality and incompleteness, which, if persistent and not addressed early on, can compromise the benefits of the system [62]. In the absence of process evaluations of the implementation of EIRs, it is likely that problems with implementation will go undetected and unaddressed and only be acknowledged when the expected benefits of EIRs are not realized. Thus, process evaluations of the initial rollout and scale-up help to mitigate such issues and are vital to inform the next stages of rollout. In this paper, we present a detailed protocol to assess the early stages of implementation of an EIR in a low-resource setting.

### Limitations

This pilot study has some limitations. This study will be conducted in only 1 province in the Lao PDR (Vientiane province) and will not be representative of the country-wide implementation. Therefore, our evaluation may fail to identify challenges in and barriers to implementation that may be present in other provinces or health facilities. However, identifying the key enablers of and barriers to EIR implementation even in this setting will help inform national scale-up and implementation by highlighting issues that may hinder implementation. This study endeavors to identify contextual factors that affect the functionalities of the EIR rather than providing a statistical representation of an entire province or country. While evaluations can be time and resource intensive, they should be conducted regularly to ensure optimal use of the system and identify opportunities for improvement.

As this is a pilot of a new protocol and approach, and outcomes of interest were not previously measured, a sample size for the health survey could not be estimated. The health facility survey may be subject to desirability bias [63], as younger health care workers might be more inclined to favor new technologies but might not always feel comfortable providing honest feedback. However, using independent enumerators with a strong background in immunization, who are trained in interviewing and rapport building, will help minimize some of these biases. Furthermore, we used purposive sampling for key informant interviews, observation, and data quality assessments, which may introduce selection bias. Due to the small sample size in this study, we were unable to compare high-performing and low-performing districts; however, such a comparison may be useful for future research. To minimize this bias, we will include a variety of stakeholders in the interviews and continue data collection until saturation is reached. Similarly, with workflow observations, there may be some observer bias; however, by maintaining distance and building a relationship of trust ahead of the interview, we will aim to minimize any influences [64]. We will also build strong relationships with in-country partners and stakeholders by establishing a technical advisory group, holding regular meetings and communication, and ensuring local communities receive all necessary information to make informed decisions to participate. For the data quality assessments, we have attempted to minimize bias by purposefully selecting 2 of the 4 sites from the low-performing districts. In addition, the hierarchical nature of the health system and reluctance to speak openly with people perceived to be in positions of authority may lead to falsely optimistic results. To mitigate this, data enumerators will be trained to build trust and rapport with participants and conduct data collection in a private location to minimize interference from others. In line with the aforementioned frameworks, it is important to acknowledge that EIR implementation is influenced by many additional health system factors, including funding, digital health infrastructure, and health literacy, which were beyond the scope of this evaluation. Despite these limitations, our protocol provides a standardized approach to evaluate EIRs in different settings.

### Conclusions

Similar robust evaluations are rarely documented and tend to focus on their findings, and guidance on how to conduct such evaluations is scarce in the literature. As a result, the findings of similar evaluations in the literature are highly varied and are often focused on a narrow aspect of the EIR. Only few studies adopt a holistic, system-wide approach. By publishing our protocol, we hope to provide countries and researchers with a standardized approach, data collection tools, and indicators that can be adapted and applied in other low-resource settings, including many of the countries that have implemented COVID-19 EIRs and are looking to integrate them for routine immunization. While the context will vary, we use the best practice research methods that are feasible to implement and can be replicated across settings. Using a standardized methodology can enable comparability of performance across different settings, keeping in mind the contextual factors.

As EIR implementation is a multiyear process, extending over 10 years, and even well-established EIRs in high-income settings constantly evolve, a single process evaluation is insufficient to ensure an effective EIR. Evaluation must be ongoing and ideally incorporated as part of the monitoring and evaluation for immunization programs.

## Appendix

Multimedia Appendix 1 Comparison of electronic immunization registries versus traditional systems.

Multimedia Appendix 2 Lao People’s Democratic Republic National Childhood Immunization Schedule, 2024.

Multimedia Appendix 3 Data collection tool for observation of immunization data workflow at health facilities.

Multimedia Appendix 4 Data collection tool for the survey of health workers at health facilities.

Multimedia Appendix 5 Data collection tool for assessment of concordance of immunization data in the electronic immunization register compared with alternative paper-based data sources.

## Abbreviations

DHIS2: District Health Information Software 2
DTP: diphtheria-tetanus-pertussis
EIR: electronic immunization register
IA2030: Immunization Agenda 2030
Lao PDR: Lao People’s Democratic Republic
LMIC: low- and middle-income country
MCHC: Maternal Child Health Centre
MoH: Ministry of Health
REDCap: Research Electronic Data Capture
UNICEF: United Nations Children’s Fund
WHO: World Health Organization
