# Supplementary material for: Strengthening Immunization Data: Protocol for the Evaluation of an Electronic Immunization Register
Source: JMIR Res Protoc. 2025 Jun 19;14:e65663. doi: 10.2196/65663 (PMC12226776; doi:10.2196/65663)
Supplement: Multimedia Appendix 1 [file resprot_v14i1e65663_app1.docx]

Multimedia Appendix 1. Comparison of electronic immunization registries versus traditional systems
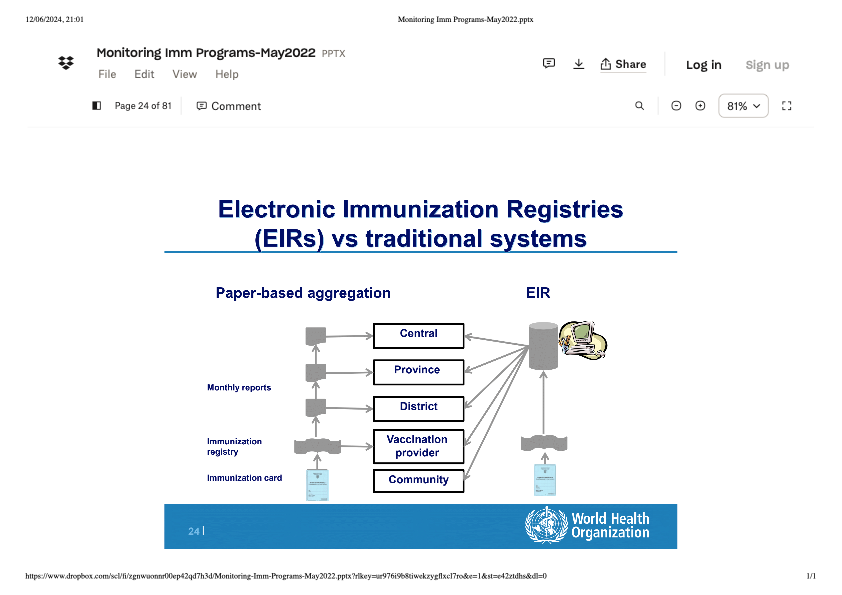


Extracted from a presentation by Danovaro-Holliday M. C. Monitoring the Immunization Programme – From a local to a global perspective. 2022.
