# Supplementary material for: Strengthening Immunization Data: Protocol for the Evaluation of an Electronic Immunization Register
Source: JMIR Res Protoc. 2025 Jun 19;14:e65663. doi: 10.2196/65663 (PMC12226776; doi:10.2196/65663)
Supplement: Multimedia Appendix 2 [file resprot_v14i1e65663_app2.docx]

Multimedia Appendix 2. Lao People’s Democratic Republic National Childhood Immunization Schedule, 2024.

| **VACCINES** | **AGES** | | | | | |
| --- | --- | --- | --- | --- | --- | --- |
|  | **Birth** | **6 weeks** | **10 weeks** | **14 weeks** | **9 to 11 months** | **12 to 18 months** |
| BCG | ✓ |  |  |  |  |  |
| Hepatitis B | ✓ |  |  |  |  |  |
| Oral polio (bOPV) |  | ✓ | ✓ | ✓ |  |  |
| DTP-HepB-Hib (Penta) |  | ✓ | ✓ | ✓ |  |  |
| Pneumococcal (PCV) |  | ✓ | ✓ | ✓ |  |  |
| Inactivated polio vaccine (IPV) |  |  |  | ✓ |  |  |
| Rotavirus vaccine RVV (Oral Rotarix in 2019) |  | ✓ | ✓ |  |  |  |
| Measles/Rubella (MR1) |  |  |  |  | ✓ |  |
| Measles/Rubella (MR2) |  |  |  |  |  | ✓ |
| Japanese Encephalitis (JE) |  |  |  |  | ✓ |  |
