# Supplementary material for: Strengthening Immunization Data: Protocol for the Evaluation of an Electronic Immunization Register
Source: JMIR Res Protoc. 2025 Jun 19;14:e65663. doi: 10.2196/65663 (PMC12226776; doi:10.2196/65663)
Supplement: Multimedia Appendix 3 [file resprot_v14i1e65663_app3.docx]

# **Multimedia Appendix 3. Data collection tool for observation of immunization data workflow at health facilities**

| **Observation details:** | |
| --- | --- |
| Date of observation |  |
| Observer name |  |
| Health facility name and district |  |
| REDCap and secondary study identifier |  |

**Instructions to data enumerator: After obtaining informed consent, please silently observe two or three vaccination encounters, and record your observations below.**

| **Checklist to be completed by the observer:** | | |
| --- | --- | --- |
| **1** | **Please mark the box that indicates what type of immunisation service is being observed.** | ☐ Fixed-site  ☐ Outreach or campaign  ☐ Other  If other, specify: [free text field] |
| **2** | **Please mark the box that best describes the immunisation service environment.** | ☐ Immunisation room (only patient and immediate family/carers)  ☐ Open plan clinic (other patients present)  ☐ Outdoors under cover  ☐ Outdoors not under cover  ☐ Other  If other, specify: [free text field] |
| **3** | **Observe two or three vaccination encounters and mark all tools used during the observation period.**  *Select ‘no’ of tool not observed or if unsure.*    ☐ Yes ☐ No Electronic Immunisation Registry (DHIS2 Tracker)  ☐ Yes ☐ No Health facility immunisation register (usually a paper-based register book ‘Child Registration Book’)  ☐ Yes ☐ No Child take-home record (Mother & Child Health ‘pink book’ or vaccination card)  ☐ Yes ☐ No Health facility tally sheet (aggregate count – usually a paper-based form)  ☐ Yes ☐ No DHIS2 tally sheet (aggregate count – digital capture)  ☐ Yes ☐ No DHIS2 child vaccination form (individual information)  ☐ Yes ☐ No Health facility service provision record (individual information)  ☐ Yes ☐ No Other recording tools not previously mentioned?  *e.g. notebooks for migrant or mobile populations*  If other, specify: [free text field]  Please note any inconsistencies of recording during the session: [free text field] | |
| **4** | **Please mark the box that indicates when vaccinations are recorded in the EIR (DHIS2 Tracker).** | ☐ Before the vaccine is administered  ☐ Immediately after the vaccine is administered and before the next patient  ☐ Tool not used during the observation period  ☐ Other  If other, specify: [free text field] |
| **5** | **Please mark the box that indicates when vaccinations are recorded in the health facility immunisation register.**  *Usually a paper-based register book ‘Child registration Book’* | ☐ Before the vaccine is administered  ☐ Immediately after the vaccine is administered and before the next patient  ☐ Tool not used during the observation period  ☐ Other  If other, specify: [free text field] |
| **6** | **Please mark the box that indicates when vaccinations are recorded in the child health record.**  *Usually the Mother & Child Health ‘pink book’ but could also be a vaccination card* | ☐ Before the vaccine is administered  ☐ Immediately after the vaccine is administered and before the next patient  ☐ Tool not used during the observation period  ☐ Other  If other, specify: [free text field] |
| **7** | **Please mark all equipment available to support recording of immunisation data.**  *Select ‘no’ of tool not observed or if unsure.*    ☐ Yes ☐ No Pen and paper  ☐ Yes ☐ No Desk or table space  ☐ Yes ☐ No Power source  ☐ Yes ☐ No Internet connection  ☐ Yes ☐ No Desktop computer  ☐ Yes ☐ No Laptop computer  ☐ Yes ☐ No Tablet e.g. iPad  ☐ Yes ☐ No Smartphone  ☐ Yes ☐ No Other  If other, specify: [free text field] | |
| **8** | **List the number of team members, and their roles, involved in the administration and recording of immunisations during the observation period.**  *e.g. a single immunisation provider only, or multiple immunisation providers, or data entry support* | [free text field] |
| **9** | **Please note down any interruptions during the observation period.**  *e.g. noise levels, internet or power outages, other clinicians, or patient interruptions etc.* | [free text field] |
| **10** | **Please note anything else observed that affected the vaccine encounter immunisation information workflow.**  *e.g. observed challenges or barriers to completing tasks, or delegation of tasks to other team members* | [free text field] |
