# Supplementary material for: Strengthening Immunization Data: Protocol for the Evaluation of an Electronic Immunization Register
Source: JMIR Res Protoc. 2025 Jun 19;14:e65663. doi: 10.2196/65663 (PMC12226776; doi:10.2196/65663)
Supplement: Multimedia Appendix 4 [file resprot_v14i1e65663_app4.docx]

# **Multimedia Appendix 4. Data collection tool for the survey of health workers at health facilities**

| **Interview details:** | |
| --- | --- |
| Date of interview |  |
| Interviewer name |  |
| Health Facility | |
| Province |  |
| District |  |
| Health Facility Name |  |
| **Details of individual being interviewed**  ***We will start by asking some professional and personal questions. These will help us to understand how your life experiences may affect your experiences with the EIR.*** | |
| Professional role | ☐ Doctor  ☐ Nurse  ☐ Doctors Assistant  ☐ Public Health Officer  ☐ Immunisation/EPI Manager  ☐ Statistician  ☐ Community Health Worker (paid)  ☐ Health Volunteer (unpaid)  ☐ Other, please specify ____________________ |
| How many years have you worked in your current position? | ☐ Less than 1 year  ☐ 1 to 4 years  ☐ 5 to 10 years  ☐ More than 10 years |
| How many years have you worked in immunisation services? | ☐ Less than 1 year  ☐ 1 to 4 years  ☐ 5 to 10 years  ☐ More than 10 years |
| How would you rate your computer skills (computer literacy)?  *i.e. that is, your level of experience using a computer* | ☐ Very good  ☐ Good  ☐ Sufficient  ☐ Poor |
| What is your current age in years? | [number - integer] |
| Do you identify as | ☐ Man  ☐ Woman  ☐ Not listed above  If not listed above, please specify ____________________  ☐ Prefer not to say |
| What is your ethnicity? | ☐ Lao  ☐ Mon Khmer  ☐ Chinese Tibetan  ☐ Other  If other, specify: ____________________  ☐ Prefer not to say |

| **1. Vaccination processes at your health facility**  ***In this section, we will ask you about vaccination services at your health facility and when you record data on vaccinations.*** | | | |
| --- | --- | --- | --- |
| 1.1 | What type of immunisation service is offered at your health facility? | ☐ Fixed-site only  ☐ Outreach or campaign  ☐ Fixed site AND outreach/campaign | |
| 1.2 | How many patient vaccination appointments does your service provide during a typical service day? | ☐ Less than 10  ☐ 10 to less than 20  ☐ 20 to less than 50  ☐ 50 to less than 100  ☐ More than 100  ☐ Other frequency  If other, please specify _______________ | |
| 1.3 | **Do you use the following tools to record immunisation data at your health facility?** | | |
|  | *Please select all that apply*   1. Electronic Immunisation Registry (DHIS2 Tracker) 2. Health facility immunisation register (paper-based register) 3. Child take-home record 4. Health facility tally sheet (aggregate count – usually paper) 5. DHIS2 tally sheet (aggregate count – digital) 6. DHIS2 Event Capture (individual information) 7. Other recording tools not previously mentioned?   *e.g. notebooks for migrant or mobile populations*  If other, please specify ___________________________ | | ☐ Yes ☐ No  ☐ Yes ☐ No  ☐ Yes ☐ No  ☐ Yes ☐ No  ☐ Yes ☐ No  ☐ Yes ☐ No  ☐ Yes ☐ No |
| **2. This set of questions is related to fixed-site immunisation services:**  **Skip these questions if fixed-site services not provided* | | | |
| 2.1 | How frequently are **fixed-site** immunisation services provided by your health facility during usual operations?    **Skip this question if fixed-site immunisation services not provided* | ☐ Almost every day (5-7 days per week)  ☐ Some days (2-4 days per week)  ☐ Weekly (1 day per week)  ☐ Fortnightly (1 day per two weeks)  ☐ Monthly (1 day per month)  ☐ Quarterly (1 day every three months)  ☐ Other frequency  If other, please specify _______________ | |
| 2.2 | What is the standard protocol for entering data entry into the EIR (DHIS2 Tracker)? | ☐ Before the vaccine is administered  ☐ Immediately after the vaccine is administered and before the next patient  ☐ By the end of the day  ☐ Within 2-3 days  ☐ At the end of the week  ☐ At the end of the month  ☐ Tool not used  ☐ Other  If other, please specify _______________ | |
| 2.3 | What do you actually do? | ☐ Before the vaccine is administered  ☐ Immediately after the vaccine is administered and before the next patient  ☐ By the end of the day  ☐ Within 2-3 days  ☐ At the end of the week  ☐ At the end of the month  ☐ Tool not used  ☐ Other  If other, please specify _______________ | |
| 2.4 | When do you enter the data into the health facility immunisation register?  *Usually a paper-based register book ‘Child Registration Book’* | ☐ Before the vaccine is administered  ☐ Immediately after the vaccine is administered and before the next patient  ☐ By the end of the day  ☐ Within 2-3 days  ☐ At the end of the week  ☐ At the end of the month  ☐ Tool not used  ☐ Other  If other, please specify _______________ | |
| 2.5 | When do you enter the data into the child take-home record?  *Usually the Mother & Child Health ‘pink book’ but could also be a vaccination card* | ☐ Before the vaccine is administered  ☐ Immediately after the vaccine is administered and before the next patient  ☐ Tool not used  ☐ Other  If other, please specify _______________ | |
| **3. Fixed-Site Immunisation Service Infrastructure**  In this section, we will ask you about the physical resources available to you to record vaccination data in the EIR. These are things like electricity, internet connection and computers or other equipment.  **Skip these questions if fixed-site services not provided* | | | |
| 3.1 | Is there a computer or tablet available for immunisation activities? (if no or unsure, go to 3.1.3) | ☐ Yes ☐ No | |
| 3.1.1 | If yes, is it located in the immunisation room? | ☐ Yes ☐ No | |
| 3.1.2 | Is this computer or tablet for use exclusively for EPI program activities? (i.e. it is only used for EPI activities) | ☐ Yes ☐ No | |
| 3.1.3 | Does your health facility have an internet connection?  *If no go to question 3.2* | ☐ Yes ☐ No | |
| 3.1.4 | Is the internet connection stable? | ☐ Stable (outages are rare)  ☐ Mostly stable (occasional outages)  ☐ Unstable (frequent outages e.g. daily) | |
| 3.1.5 | Does your health facility have access to funding for the internet? | ☐ Always  ☐ Usually  ☐ Frequently not available  ☐ Not available | |
| 3.1.6 | Where does the funding come from? |  | |
| 3.2 | What type of power source is used at your health facility? | ☐ Electricity  ☐ Generator  ☐ Other  If other, please specify _______________ | |
| 3.2.1 | Does your facility ever experience power outages?  If no, go to question 3.3 | ☐ Yes ☐ No ☐ Unsure | |
| 3.2.2 | If yes, how often? | ☐ Rarely e.g. 1-2 times per year  ☐ Occasional e.g. 1-2 times per month  ☐ Often e.g. every week or daily | |
| 3.2.3 | Is there a plan or protocol for how to register immunisations during power outages? | ☐ Yes ☐ No ☐ Unsure  If yes, please specify _______________ | |
| 3.3 | In your opinion, is there sufficient infrastructure to support efficient recording of vaccination data for fixed site services? e.g. computers, internet, power, other items | ☐ Sufficient (adequate)  ☐ Often insufficient  ☐ Insufficient | |
| 3.4 | How do you think infrastructure could be improved? |  | |
| **4. This set of questions is related to outreach/campaign immunisation services:**  **Skip these questions if outreach/campaign services not provided* | | | |
| 4.1 | How frequently are **outreach**/**campaign** immunisation services provided by your health facility during usual operations? | ☐ Almost every day (5-7 days per week)  ☐ Some days (2-4 days per week)  ☐ Weekly (1 day per week)  ☐ Fortnightly (1 day per two weeks)  ☐ Monthly (1 day per month)  ☐ Quarterly (1 day every three months)  ☐ Other frequency  If other, please specify _______________ | |
| 4.2 | What is the standard protocol for entering data into the EIR (DHIS2 Tracker)? | ☐ Before the vaccine is administered  ☐ Immediately after the vaccine is administered and before the next patient  ☐ By the end of the day  ☐ Within 2-3 days  ☐ At the end of the week  ☐ At the end of the month  ☐ Tool not used  ☐ Other  If other, please specify _______________ | |
| 4.3 | What do you actually do? | ☐ Before the vaccine is administered  ☐ Immediately after the vaccine is administered and before the next patient  ☐ By the end of the day  ☐ Within 2-3 days  ☐ At the end of the week  ☐ At the end of the month  ☐ Tool not used  ☐ Other  If other, please specify _______________ | |
| 4.4 | When do you enter the data into the health facility immunisation register?  *Usually a paper-based register book ‘Child Registration Book’* | ☐ Before the vaccine is administered  ☐ Immediately after the vaccine is administered and before the next patient  ☐ By the end of the day  ☐ Within 2-3 days  ☐ At the end of the week  ☐ At the end of the month  ☐ Tool not used  ☐ Other  If other, please specify _______________ | |
| 4.5 | When do you enter the data into the child take-home record?  *Usually the Mother & Child Health ‘pink book’ but could also be a vaccination card* | ☐ Before the vaccine is administered  ☐ Immediately after the vaccine is administered and before the next patient  ☐ Tool not used  ☐ Other  If other, please specify _______________ | |
| **5. For outreach/campaign immunisation services:**  In this section, we will ask you about the physical resources available to you to record vaccination data in the EIR. These are things like electricity, internet connection and computers or other equipment.  **Skip these questions if outreach/campaign services not provided* | | | |
| 5.1 | Is there a computer or tablet available for immunisation activities?  *If no, go to question 5.1.2* | ☐ Yes ☐ No | |
| 5.1.1 | Is this computer or tablet for use exclusively for EPI program activities? | ☐ Yes ☐ No | |
| 5.1.2 | Is there an internet connection available?  *If no, go to question 5.2* | ☐ Yes ☐ No | |
| 5.1.3 | If yes, is the internet connection stable? | ☐ Stable (outages are rare)  ☐ Mostly stable (occasional outages)  ☐ Unstable (frequent outages e.g. daily) | |
| 5.1.4 | Does your health facility have access to funding for internet? | ☐ Always  ☐ Usually  ☐ Frequently not available  ☐ Not available | |
| 5.1.5 | Where does the funding come from? | *e.g. provincial government, donor, personal, health facility,, MCHC* | |
| 5.2 | Is there a power source available?  *If no, go to question 5.3* | ☐ Yes ☐ No | |
| 5.2.1 | What type of power source is available? | ☐ Electricity  ☐ Generator  ☐ Other  If other, please specify _______________ | |
| 5.2.2 | If yes, do power outages occur? | ☐ Yes ☐ No | |
| 5.2.3 | If yes, how often? | ☐ Rarely e.g. 1-2 times per year  ☐ Occasional e.g. 1-2 times per month  ☐ Often e.g. every week or daily | |
| 5.2.4 | Is there a plan or protocol for how to register immunisations during power outages? | ☐ Yes ☐ No ☐ Unsure  If yes, specify: _______________ | |
| 5.3 | In your opinion, is there sufficient infrastructure to support efficient recording of immunisation data? e.g. computers, internet, power, other items | ☐ Sufficient (adequate)  ☐ Often insufficient  ☐ Insufficient | |
| 5.4 | How do you think infrastructure could be improved? | _______________ | |
| **6. Data use and management**  ***In this section, we will ask you about what immunisation data is available to you and how you use this data.*** | | | |
| 6.1 | Do you have information on number of newborns in your area?  *e.g. a facility birth book, or a report of births* | ☐ Yes ☐ No ☐ Unsure  If yes, please specify _______________ | |
| 6.2 | Is there a process to track people who are overdue for immunisation using data from the health facility? (if no or unsure, go to 6.5) | ☐ Yes ☐ No ☐ Unsure | |
| 6.3 | If yes, what tool is used? |  | |
| 6.4 | In your opinion, is this tool effective in supporting follow-up with children who have not received their vaccines according to the schedule? | ☐ Yes ☐ No  Please specify why it is or isn’t effective _______________ | |
| 6.5 | Are you able to access coverage estimates that relate to your target population? | ☐ Yes ☐ No | |
| 6.6 | Do you have data on children who have dropped out? | ☐ Yes ☐ No | |
| 6.7 | Is there a regular meeting to discuss immunisation data analyses and results in the health facility? | ☐ Yes ☐ No ☐ Unsure | |
| 6.8 | Is there a way for you to seek help if you experience problems or issues with data in the EIR (DHIS2 Tracker)? | ☐ Yes ☐ No ☐ Unsure  If yes, please specify _______________ | |
| 6.9 | Is there a process for dealing with errors in the EIR (DHIS2 Tracker)?  *e.g. when it may be necessary to change/update the information in the system* | ☐ Yes ☐ No ☐ Unsure  If yes, please describe the process _______________________________ | |
| **7. Workforce**  ***In this section, we will ask you about the capacity of staff in your health facility to record immunisation data using the EIR.*** | | | |
| 7.1 | How many staff members work in immunisation at the health facility? | _______________ | |
| 7.2 | How many of these staff can competently enter data into the EIR (DHIS2 Tracker)? | _______________ | |
| 7.3 | In your opinion, how easy is it to learn to use the EIR (DHIS2 Tracker)? | ☐ Very easy  ☐ Somewhat easy  ☐ Not easy  ☐ Very difficult | |
| 7.4 | In your opinion, have staff received sufficient training on use of the EIR (DHIS2 Tracker)? | ☐ Sufficient (adequate)  ☐ Somewhat sufficient  ☐ Insufficient  ☐ No training | |
| 7.5 | In your opinion, how has the EIR (DHIS2 Tracker) affected the workload of your team? | ☐ Reduced workload  ☐ Workload stayed the same  ☐ Increased workload  ☐ Unsure | |
| 7.6 | In your opinion, what specific EIR (DHIS2 Tracker) training do you think would be useful for staff at your health facility? | _______________ | |
| **8. User experience and perception**  ***In this section, we will ask you about how you feel about using the EIR.*** | | | |
| 8.1 | How easy do you find the EIR to use? | ☐ Very easy  ☐ Somewhat easy  ☐ Not easy  ☐ Very difficult | |
| 8.2 | How much faster does the EIR make immunisation data capture compared to using the paper-based register? | ☐ Much faster  ☐ A little faster  ☐ Not faster  ☐ Much slower | |
| 8.3 | How much easier does the EIR make the vaccination process compared to using a paper-based register? | ☐ Much easier  ☐ Somewhat easier  ☐ Not easier  ☐ Harder | |
| 8.4 | How safe/secure is the immunisation data in the EIR? | ☐ Very safe  ☐ Somewhat safe  ☐ Not safe  ☐ Very unsafe | |
| 8.5 | Overall, how would you rate your satisfaction with the EIR (DHIS2 Tracker)? | ☐ Very satisfied  ☐ Satisfied  ☐ Unsatisfied  ☐ Very unsatisfied | |
| 8.6 | Are there other aspects of using the EIR that have made you satisfied or unsatisfied? | _______________ | |
| **9. Perception of ‘information quality’ at health facility level**  ***In this section, we will ask you about how useful you think data from the EIR is.***  *On a scale of 0 to 10 with 0 being the lowest (worst) score and 10 being the highest (best) score, please respond to the following statements* | | | |
| 9.1 | How satisfied are you with the **accuracy** of immunisation records in the EIR? | ☐ Very satisfied  ☐ Satisfied  ☐ Unsatisfied  ☐ Very unsatisfied | |
| 9.2 | How satisfied are you with the **completeness** of immunisation records in the EIR? | ☐ Very satisfied  ☐ Satisfied  ☐ Unsatisfied  ☐ Very unsatisfied | |
| 9.3 | How satisfied are you with the **timeliness** of immunisation data capture in the EIR? | ☐ Very satisfied  ☐ Satisfied  ☐ Unsatisfied  ☐ Very unsatisfied | |
| 9.4 | How easy is it to access the information in the EIR when you need it? | ☐ Very easy  ☐ Somewhat easy  ☐ Not easy  ☐ Very difficult | |
| 9.5 | How satisfied are you that the EIR provides the information you need to do your job? | ☐ Very satisfied  ☐ Satisfied  ☐ Unsatisfied  ☐ Very unsatisfied | |
| 9.6 | Overall, how useful do you think that the data in EIR is? | ☐ Very useful  ☐ Useful  ☐ Not very useful  ☐ Not at all useful | |
| 9.7 | Is there any information not available in the EIR that you think would be useful?  *For example, data on ethnicity, language, or disability?* |  | |
| 9.8 | Why do you think the data in the EIR is, or is not, useful? |  | |
| 9.9 | Have you found any challenges with EIR information/data quality not previously mentioned? | _______________ | |
| **10. Additional relevant information not captured previously** | | | |
| 10.1 | Is there anything else you would like to add in relation to the EIR? | _______________ | |
