# Supplementary material for: Strengthening Immunization Data: Protocol for the Evaluation of an Electronic Immunization Register
Source: JMIR Res Protoc. 2025 Jun 19;14:e65663. doi: 10.2196/65663 (PMC12226776; doi:10.2196/65663)
Supplement: Multimedia Appendix 5 [file resprot_v14i1e65663_app5.docx]

**Multimedia Appendix 5. Data collection tool for assessment of concordance of immunization data in the electronic immunization register compared with alternative paper-based data sources**

**Instructions:** Columns A - B (number of vaccines) to be completed at the Health Facility level.

| **Assessment details** | |
| --- | --- |
| Date of assessment |  |
| Assessor name |  |
| Health Facility |  |
| District |  |

| **Month & Year** | **Vaccine and dose** | **A**  **Health facility register (<5y)** | **B**  **Monthly immunisation report** | **C**  **Electronic Immunisation Register** |
| --- | --- | --- | --- | --- |
| **Month 1 (Nov 2023):**  **[month & year]** | Hep B birth dose |  |  |  |
|  | Penta 1 |  |  |  |
|  | MCV 1 |  |  |  |
| **Month 2 (Dec 2023):**  **[month & year]** | Hep B birth dose |  |  |  |
|  | Penta 1 |  |  |  |
|  | MCV 1 |  |  |  |
| **Month 3 (Jan 2024):**  **[month & year]** | Hep B birth dose |  |  |  |
|  | Penta 1 |  |  |  |
|  | MCV 1 |  |  |  |

1. Meet with the facility manager to provide information on data concordance and its purpose.
2. Request data for November 2023, December 2023, and January 2024 at the health facility level.
3. Review register for each month, and extract data for number of Hep B, number of Pentavalent (Diphtheria, Pertussis, Tetanus, Hepatitis B and Hib) and measles-containing vaccine (MCV1)
4. Request data from monthly report for the <5 year old routine vaccination data for November 2023, December 2023, and January 2024. The monthly immunisation data might be requested from the District Office or from the Provincial EPI manager for that health facility.
5. Once all facilities are completed, request for data from the EIR from the MOH and/or MCHC.
